# A quantitative clinical evaluation of simultaneous reconstruction of attenuation and activity in time-of-flight PET

**DOI:** 10.1186/s12880-023-00987-7

**Published:** 2023-02-27

**Authors:** Haiqiong Zhang, Jingnan Wang, Nan Li, Yue Zhang, Jie Cui, Li Huo, Hui Zhang

**Affiliations:** 1 Department of Nuclear Medicine, State Key Laboratory of Complex Severe and Rare Diseases, Beijing Key Laboratory of Molecular Targeted Diagnosis and Therapy in Nuclear Medicine, Peking Union Medical College Hospital, Chinese Academy of Medical Sciences, Beijing, 100730 China; 2 Medical Science Research Center (MRC), Peking Union Medical College Hospital, Chinese Academy of Medical Sciences, Beijing, 100730 China; 3 SinoUnion (Beijing) Healthcare Technologies Co., Ltd, Beijing, 100082 China; 4 Department of Biomedical Engineering, Tsinghua University, Beijing, 100084 China

**Keywords:** TOF-PET, MLAA, CT-OSEM, Quantitative evaluation

## Abstract

**Background:**

The maximum likelihood activity and attenuation (MLAA) reconstruction algorithm has been proposed to jointly estimate tracer activity and attenuation at the same time, and proven to be a promising solution to the CT attenuation correction (CT-AC) artifacts in PET images. This study aimed to perform a quantitative evaluation and clinical validation of the MLAA method.

**Methods:**

A uniform cylinder phantom filled with ^18^F-FDG solution was scanned to optimize the reconstruction parameters for the implemented MLAA algorithm. 67 patients who underwent whole-body ^18^F-FDG PET/CT scan were retrospectively recruited. PET images were reconstructed using MLAA and clinical standard OSEM algorithm with CT-AC (CT-OSEM). The mean and maximum standardized uptake values (SUVmean and SUVmax) in regions of interest (ROIs) of organs, high uptake lesions and areas affected by metal implants and respiration motion artifacts were quantitatively analyzed.

**Results:**

In quantitative analysis, SUVs in patient’s organ ROIs between two methods showed *R*^2^ ranging from 0.91 to 0.98 and *k* ranging from 0.90 to 1.06, and the average SUVmax and SUVmean differences between two methods were within 10% range, except for the lung ROI, which was 10.5% and 16.73% respectively. The average SUVmax and SUVmean differences of a total of 117 high uptake lesions were 7.25% and 7.10% respectively. 20 patients were identified to have apparent respiration motion artifacts in the liver in CT-OSEM images, and the SUVs differences between two methods measured at dome of the liver were significantly larger than measured at middle part of the liver. 10 regions with obvious metal artifacts were identified in CT-OSEM images and the average SUVmean and SUVmax differences in metal implants affected regions were reported to be 52.90% and 56.20% respectively.

**Conclusions:**

PET images reconstructed using MLAA are clinically acceptable in terms of image quality as well as quantification and it is a useful tool in clinical practice, especially when CT-AC may cause respiration motion and metal artifacts. Moreover, this study also provides technical reference and data support for the future iteration and development of PET reconstruction technology of SUV accurate quantification.

## Background

Dual modality positron emission tomography (PET)/computed tomography (CT) has wide applications in various clinical disciplines such as oncology, neurology and cardiology. During a PET/CT scan, the CT images are used to provide anatomical information as well as to perform attenuation correction (AC) for the PET, which requires the CT and PET images to be well-aligned. CT images are acquired considerably fast. PET images, however, are typically acquired while the patient is free breathing due to the length of the PET scan time. This may lead to spatial mismatch between CT and PET images, especially for those organs and anatomical regions affected by involuntary motions such as heart beat and respiration. To solve this mismatch, different strategies such as gated acquisitions have been proposed [1, 2], but this method generally needs more acquisition time for adequate statistics. Additional difficulties with CT-based AC arise when contrast agents are used for the CT imaging [3], or when the patient carries metal implants [4], or when the patient body extends out of the trans-axial field-of-view covered by the CT [5, 6].

In recent years, the joint estimation of radiotracer activity and attenuation information in time-of-flight PET (TOF-PET) have been considered to achieve quantitative analysis in the scenarios of mismatch between CT and PET images, such as maximum likelihood reconstruction of attenuation and activity (MLAA) [7–9], maximum likelihood attenuation correction factors (MLACF) [10–12], maximum likelihood reconstruction of activity and registration of attenuation (MLRR) [13]. Compared to MLAA, images reconstructed using MLACF may be noisier due to the lack of the consistent attenuation factors [11, 14], and MLACF as well as MLRR are relatively slower in convergence [10, 15, 16]. Hence, MLAA was chosen in this study to reconstruct PET images without using CT based attenuation correction.

The basic idea of MLAA is to simultaneously reconstruct the tracer distribution and the attenuation image using the PET emission data only. Early attempts suffered from severe “cross-talk” between the estimated activity and attenuation distributions because the solution to the simultaneous estimation is not unique in the absence of prior information [17]. Recent studies, however, reveal that using TOF information, the ill-posed joint estimation problem can reach a stable solution and the local cross-talk is expected to be eliminated, enabling full exploitation of MLAA [18]. A potential problem of the joint estimation is that the derived attenuation image cannot be determined unambiguously, but rather to within a constant scaling factor, which may cause problems in specific clinical practices where quantitative PET imaging is required. The scale problem was addressed by including a priori knowledge of the attenuation coefficients such as the typical attenuation values in tissues [19].

Existing studies have demonstrated that the MLAA joint estimation algorithms help to reconstruct more accurate AC maps for PET/CT. The MLAA method was also evaluated in cardiac applications with 14 ^13^N-ammonia PET/CT perfusion studies [20] and 12 healthy volunteers with CO_2_ stress in ^82^Rb PET/CT respiratory gated imaging, and the results showed that the MLAA joint estimation was able to remove the possible PET/CT mismatch [21]. The MLAA algorithm was compared to the maximum likelihood expectation maximization (MLEM) reconstructions with CT-based AC on 23 torso ^18^F-FDG patient scans and the joint estimation results were found to be within clinical acceptable accuracy [19]. In addition, compared to the gold-standard CT-derived attenuation map, deep learning (DL) and convolutional neural network (CNN) were applied to predict the CT attenuation map from MLAA pre-computed results in quantitative evaluations [22, 23]. Of course, there are also corresponding studies to improve the aspect of AC in PET/MR [24–29].

The reported investigations focused on research tracers and small samples of ^18^F-FDG clinical study. However, comprehensive and quantitative evaluations for MLAA clinical validation using large number of ^18^F-FDG patients are still needed before it can be widely used in the PET/CT clinical practice, especially in the case of metal implants and motion-related artifacts in the liver dome. In this contribution, we aimed at performing a quantitative analysis of the reconstructed PET images obtained with the MLAA algorithm on both a phantom scan and 67 clinical patient scans, compared to the clinical standard 3D TOF-ordered subsets expectation maximization (OSEM) with CT-based AC.

## Methods

### The MLAA algorithm

The study was carried out at Peking Union Medical College Hospital (PUMCH) using the MLAA reconstruction program installed in a stand-alone ARW workstation (SinoUnion Healthcare, Beijing, China) for clinical research purposes. The MLAA algorithm was implemented similar to that described in [7], which makes alternated updates to the activity and attenuation images in each MLAA iteration using the TOF based MLEM and maximum-likelihood for transmission (MLTR) algorithms. Both the MLEM and MLTR components were accelerated with ordered subsets. The scale problem of the implemented MLAA was fixed by imposing a total tracer activity prior during the MLAA iterations [18]. The total tracer activity was computed by the MLEM reconstruction in advance with CT-based attenuation image. Some motion-affected regions (e.g. the apex and base of the lung, the dome of liver) and high density implants regions were excluded in the estimation by thresholding CT images to minimize the effect of data correction inconsistencies. The algorithm was initialized with uniform activity and attenuation in the field of view. The final activity reconstruction was post-smoothed with a Gaussian filter of 4.5 mm full width at half maximum (FWHM). The pre-computed single-scatter estimation was performed based on the CT as the additive contribution.

### Phantom studies

A phantom study was firstly performed to assess the performance of the implemented MLAA algorithm as well as to determine the recommended reconstruction parameters setting for the clinical applications. A uniform cylinder phantom (diameter: 20 cm, height 25 cm) filled with 120.60 MBq (3.26 mCi) ^18^F-FDG solution was scanned on a PoleStar m680 PET/CT scanner with 418.5 ps TOF resolution (SinoUnion Healthcare, Beijing, China). The activity concentration in the phantom equals to 15.36 kBq/cc at time of scanning, which approximately corresponds to the average in the clinically relevant activity concentration range of 10–20 kBq/cc. Online reconstruction was performed using the TOF-OSEM algorithm with CT-based AC (CT-OSEM) with 3 iterations of 10 subsets, and 4.5-mm FWHM Gaussian post-filtering. The image was 192 × 192 matrix with a pixel size of 3.15 × 3.15 mm. The reconstructed images were referred to as CT-OSEM images herein. The acquired listmode data was then transferred to the ARW to perform the off-line MLAA reconstruction and the reconstructed images were referred to as MLAA images. To study the behavior of the MLAA algorithm, the activity and attenuation reconstructions were obtained with 1–7 iterations of 10 subsets respectively. The reconstructed MLAA images with different iterations were compared with the CT-OSEM images. For quantitative analysis, a cylindrical region of interests (ROI) with 15 cm in diameter and 12 cm in height was placed at the center of the phantom and the mean values of the reconstructed activity concentration in the ROI were compared.

### Patient studies

Patient studies were carried out at PUMCH and 60 patients underwent whole-body PET/CT scan in clinical routine examination were recruited retrospectively. In addition, 7 patients with metal implants such as heart pacemaker and denture were also included. All patients are ranged from 18 to 70 years old. ^18^F-FDG PET/CT images were acquired supine from skull base to middle thigh level using the m680 PET/CT with an injection dose of 3.70–5.55 MBq/kg (0.1–0.15 mCi/kg) and scanned 40–60 min later. The PET data were acquired in three-dimensional acquisition mode, with 2 min per bed position and 5–6 beds per patient. The CT scans were performed at 120 kV, 160 effective mA. CT-OSEM images in 192 × 192 matrix with a pixel size of 3.15 × 3.15 mm were reconstructed with 3 iterations of 10 subsets, and 4.5 mm FWHM Gaussian post-filtering. The acquired listmode data were transferred to the ARW and MLAA images were also reconstructed with 3 iterations of 10 subsets, and 4.5 mm FWHM Gaussian post-filtering.

All the images were reviewed on a MIM workstation (MIM Software Beijing, China). Both visual comparisons and quantitative evaluations were performed on the two types of reconstructed images. Visual comparisons were performed by two experienced physicians blinded to the reconstruction type. In the quantitative analysis, the mean and maximum standardized uptake values (SUVmean and SUVmax) in different organs including the bladder, liver, spleen, heart, lung, vertebrae and muscle were compared between the MLAA and CT-OSEM reconstructed images. ROIs were manually drawn in these organs on CT-OSEM reconstructed images, and copied to MLAA reconstructed images. For heart regions, myocardium with visible ^18^F-FDG uptake were selected as ROIs. The dome of liver regions that could be affected by breathing were avoided in the ROIs selection. Tumor and inflammatory lesions with high local tracer uptake were also identified and their SUV measurements were quantitatively analyzed. Up to 5 tumor or inflammatory lesions with relatively large size per patient were selected to reduce partial volume effect. For these high uptake lesions, ROIs were also drawn on CT-OSEM reconstructed images, and copied to MLAA reconstructed images, selected by 40% threshold delineation method. Areas affected by metal artifacts were delineated on the two reconstructed images in 7 patients with metal implant. In the comparative analysis, the correlations between the SUVs in each ROI measured in the MLAA and CT-OSEM reconstructed images were studied respectively. The differences in the SUV measurements in each ROI per patient were also calculated. The CT-OSEM reconstruction was considered as the reference and the absolute difference between the SUVs in each patient was defined as:

1 $${\text{SUV difference }}\left( {\text{\% }} \right) = \left| {\frac{{{\text{SUV }}\left( {\text{MLAA reconstruction}} \right) - {\text{SUV }}\left( {{\text{CT}} - {\text{OSEM reconstruction}}} \right)}}{{{\text{SUV }}\left( {{\text{CT}} - {\text{OSEM reconstruction}}} \right)}}} \right|{ } \times { }100{\%}$$

To evaluate the behavior of the two methods in case of motion, patients with apparent respiration motion artifacts in the dome of the liver were identified in CT-OSEM images. For each of these patients, two ROIs were manually drawn in the liver in the MLAA reconstructed images, one located in the dome of the liver and the other in the middle part of the liver. The ROIs were chosen to avoid any suspected region of high local tracer uptake. The ROIs were copied to the CT-OSEM reconstructed images and the SUVs in these ROIs were quantitatively analyzed.

## Results

### Phantom studies

**Fig. 1:**
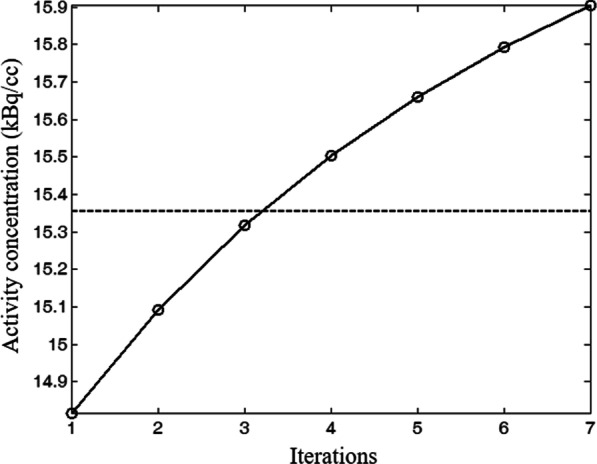
Dependency of reconstructed activity concentration on the iteration number for the MLAA reconstruction. The dash line represents the theoretic activity concentration

**Fig. 2:**
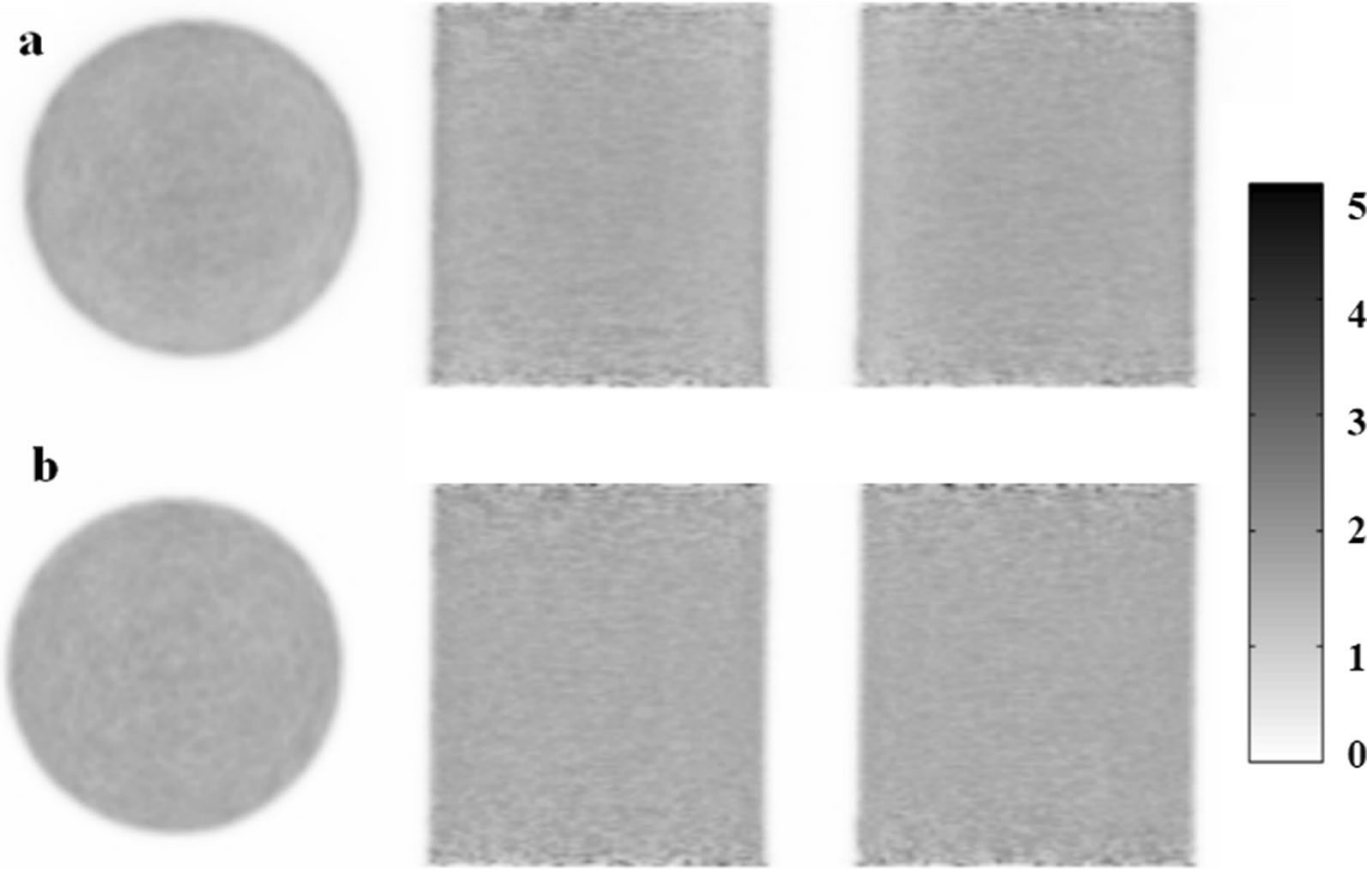
Transaxial (left), coronal (center) and sagittal (right) view of the cylinder phantom images reconstructed using **a** MLAA and **b** CT- OSEM both with 3 iterations and 10 subsets

Figure 1 shows the dependency of mean values of the reconstructed activity concentration on the iteration times in the MLAA images. The dash line represents the theoretic activity concentration of 15.36 kBq/cc. For MLAA reconstructions, the result obtained with 3 iterations is the closest to the theoretic value, with a measured mean activity concentration of 15.32 kBq/cc. For CT-OSEM reconstructions, the mean value was measured to be 15.35 KBq/cc. Visual inspection of the MLAA images with 3 iterations suggested satisfactory image quality, with no evident artifact and difference in the MLAA images compared to the CT-OSEM images, as shown in Fig. 2. Based on the phantom study results, an iteration number of 3 was chosen for the MLAA reconstructions in the following patient studies.

### Patient studies

#### Clinical image quality assessment

**Fig. 3:**
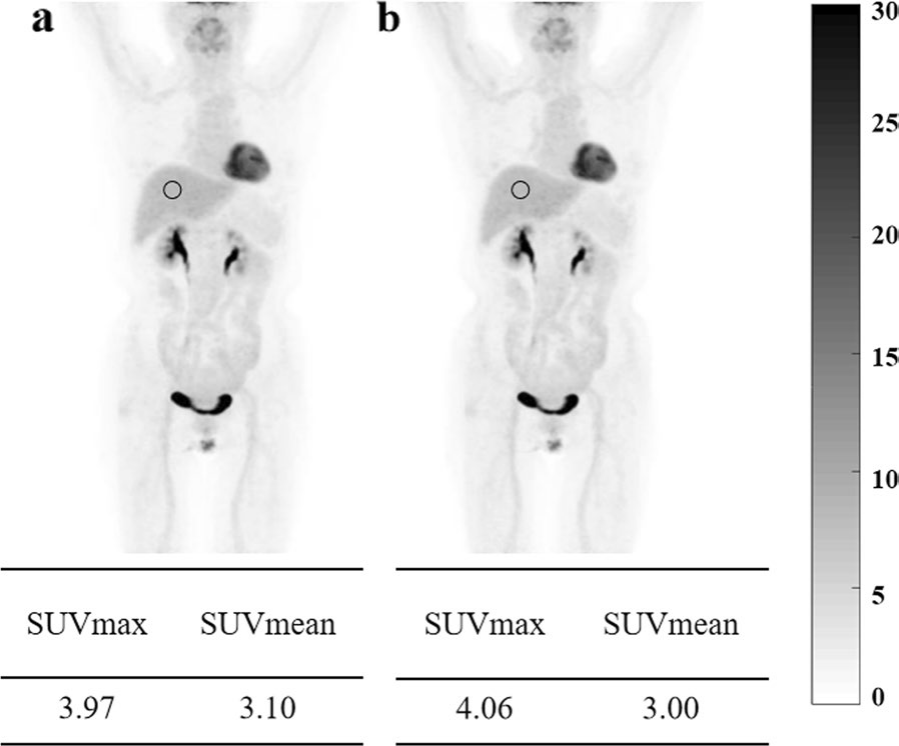
MIP of a patient whole-body ^18^F-FDG image obtained with MLAA reconstruction (**a**) and CT-OSEM reconstruction (**b**). The SUVs of the ROIs on the liver are also shown in the figure

**Fig. 4:**
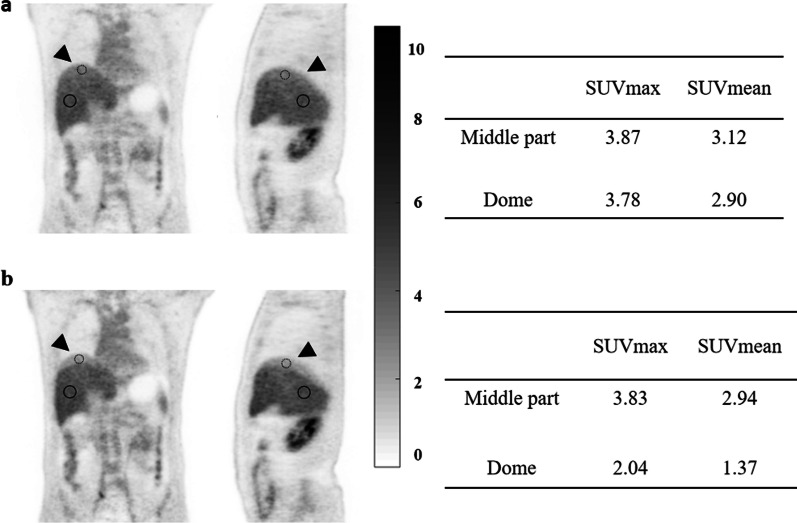
Coronal (left) and sagittal (right) view of a patient PET image obtained with MLAA reconstruction (**a**) and CT-OSEM reconstruction (**b**). Motion caused CT AC artifacts are indicated by the arrows. The SUVs in the ROIs in the middle part and the dome of the liver are also shown in the figure

For the 60 patients enrolled in the study, no evident motion-caused AC mismatch artifacts were found in the CT-OSEM images in 40 patients. Figure 3 shows a representative MIP image of a patient reconstructed using the MLAA algorithm and the CT-OSEM algorithm respectively. No obvious artifacts as well as differences were found in the two types of reconstructions for this patients and the liver SUVmax and SUVmean measurements are also comparable, with 3.97 and 3.10 respectively in the MLAA images and 4.06 and 3.00 respectively in the CT-OSEM images. AC mismatch artifacts caused by motion were found in the remaining 20 patients by reviewing the CT-OSEM images. For these patients, visual inspection of the MLAA images suggested similar or better image quality compared to the CT-OSEM images, and no obvious motion artifacts were seen in the reconstructed MLAA images. Figure 4 shows one such case where obvious AC mismatch artifact was found in the dome of the liver in the CT-OSEM images while no artifact was seen in the MLAA images. SUVs at two different locations, as shown in Fig. 4, were compared. In the CT-OSEM images, the SUVmax and SUVmean were measured to be 2.04 and 1.37 respectively in the dome of the liver, and 3.83 and 2.94 respectively in the middle part of the liver. As a comparison, in the MLAA images, the SUVmax and SUVmean were measured to be 3.78 and 2.90 respectively in the dome of the liver, and 3.87 and 3.12 respectively in the middle part of the liver, suggesting a more uniform uptake distribution in the liver region in the MLAA images.

**Fig. 5:**
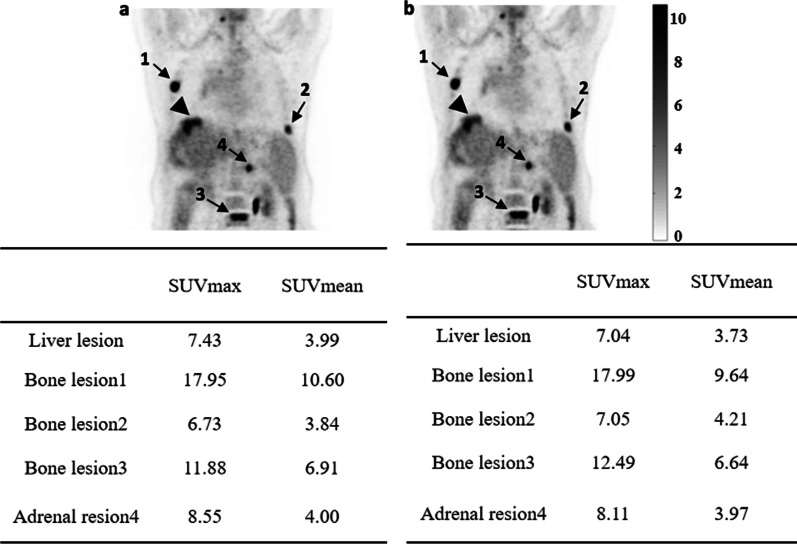
Coronal view of a patient PET image obtained with MLAA reconstruction (**a**) and CT-OSEM reconstruction (**b**). Lesions located in the liver dome are indicated by arrows and other non-motion affected lesions are also observed. The lesion SUVs are also shown in the figure

**Fig. 6:**
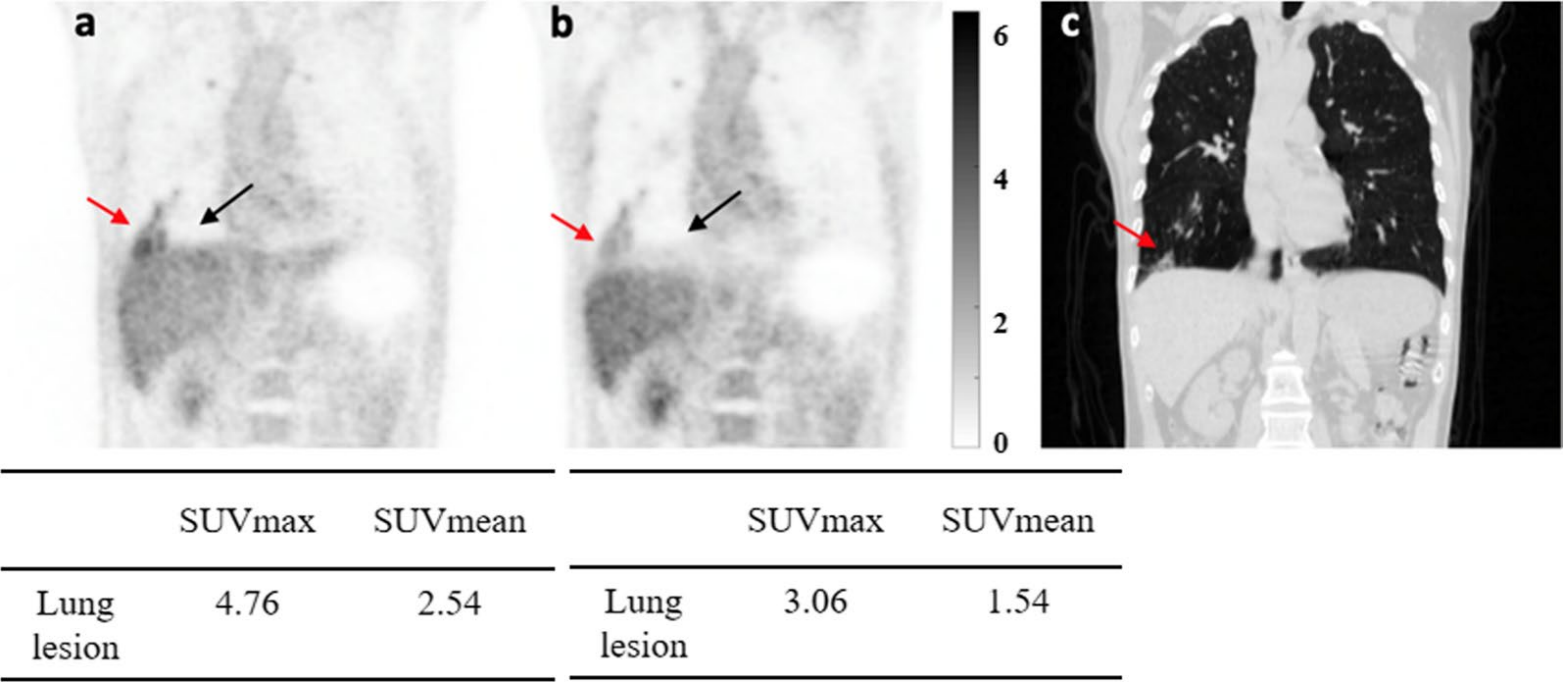
Coronal view of a patient having a lesion located at the lung base which is affected by respiratory motion. PET images obtained with MLAA reconstruction (**a**), CT-OSEM reconstruction (**b**) and CT image are shown in (**c**). Lesion is indicated by red arrows and motion caused CT AC artifacts in the liver dome is indicated by black arrows. The SUVs of the lesion are also shown in the figure

A total of 117 tumor or inflammatory lesions with relatively high uptakes were delineated in the 60 patients enrolled in the study. Visual inspection of all the lesions found no apparent difference between the two reconstruction methods except for one case. Figure 5 shows a representative case in which 5 lesions located in the liver dome, bones and adrenal gland were identified respectively . For these lesions, SUVs were also measured and shown in the figure, and both the SUVmax and SUVmean results were comparable between the two reconstruction methods. Figure 6 shows the one case with noticeable difference in the lesion between the MLAA and CT-OSEM images. The lesion is located at the lung base and due to the respiratory motion, obvious difference can be observed by comparing the two reconstructed images. The SUVmax and SUVmean of the lesion were measured to be 3.06 and 1.54 respectively in the CT-OSEM images, and measured values increased to 4.76 and 2.54 respectively in the MLAA images.

**Fig. 7:**
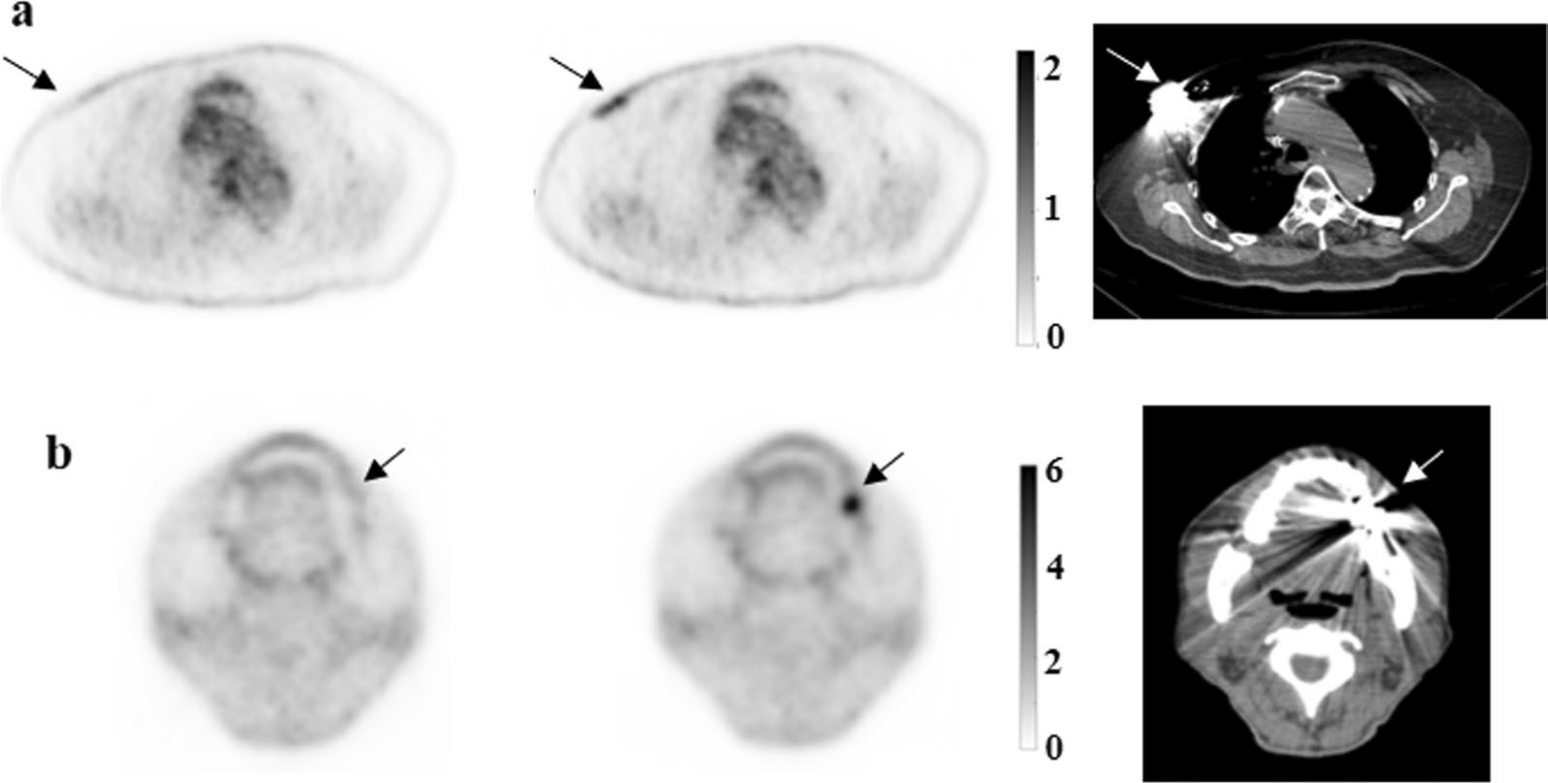
Results of a patient with a heart pacemaker (**a**) and a patient with a denture (**b**). Images shown are the transaxial view of the PET images obtained with MLAA reconstruction (left) and CT-OSEM reconstruction (center). The CT images (right) are also shown. Arrows indicate a false positive reading in the CT-OSEM images

For the 7 patients with metal implants, a total of 10 regions with metal artifacts were identified in the CT-OSEM images. These regions exhibited increased uptake, which in some cases lead to false positive readings in the PET images, as shown in Fig.7. However, no such false positive readings were observed in the MLAA images for these patients.

#### Quantitative analysis of the clinical images

**Fig. 8:**
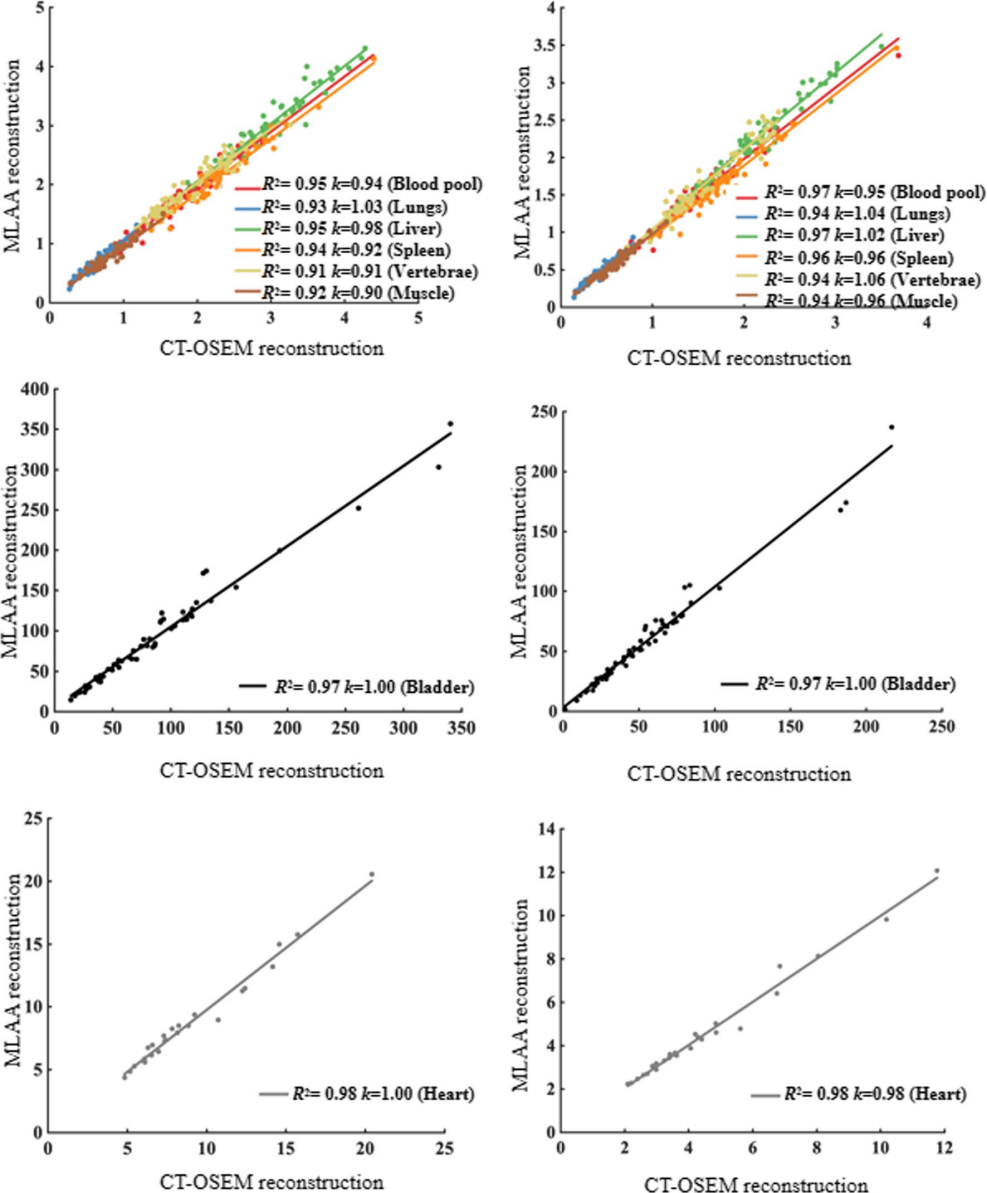
Linear regression analysis of per patient (left) SUVmax and (right) SUVmean measurements between MLAA images and CT-OSEM images in different organ ROIs. *R*^2^ is the square of correlation coefficient, *k* is the fitting slope

In the quantitative analysis, we investigated the correlations between the SUV measurements in different organs in the MLAA and CT-OSEM reconstructed images. Figure 8 shows the linear regression and linear correlations between the SUV measurements obtained by these two methods in different organ ROIs. The SUVs of bladder region were relatively high and were plotted in separate graphs. Besides, 25 patients in this study showed myocardial ^18^F-FDG uptake and the results were also plotted in separate graphs. In the linear fittings of SUVmax, the fitting slopes are in the range of 0.90–1.03 while for SUVmean, the fitting slopes are in the range of 0.95–1.06, showing good consistency between the measurements obtained in the MLAA images and CT-OSEM images. Good correlations in the SUV values per patient in each organ ROIs were also observed with a square of the correlation coefficient *R*^2^ ranging from 0.91 to 0.98. The average SUVmax and SUVmean differences in each organ ROIs for all the patients, reported as mean ± standard deviation, were calculated and the results were listed in Table 1.

**Table 1:** The average SUV differences between MLAA images and the reference CT-OSEM images in each organ ROI. Data represents the mean ± standard deviation

	SUVmax difference (%)	SUVmean difference (%)
Bladder	8.75 ± 8.13	8.82 ± 7.51
Vertebrae	5.48 ± 4.51	6.46 ± 6.18
Muscle	7.39 ± 5.89	5.86 ± 5.55
Liver	3.91 ± 3.99	6.19 ± 3.78
Spleen	8.03 ± 5.18	6.06 ± 4.88
Lungs	10.58 ± 7.38	16.73 ± 8.58
Blood pool	5.34 ± 4.52	4.62 ± 4.18
Heart	5.32 ± 3.64	4.18 ± 3.29

**Fig. 9:**
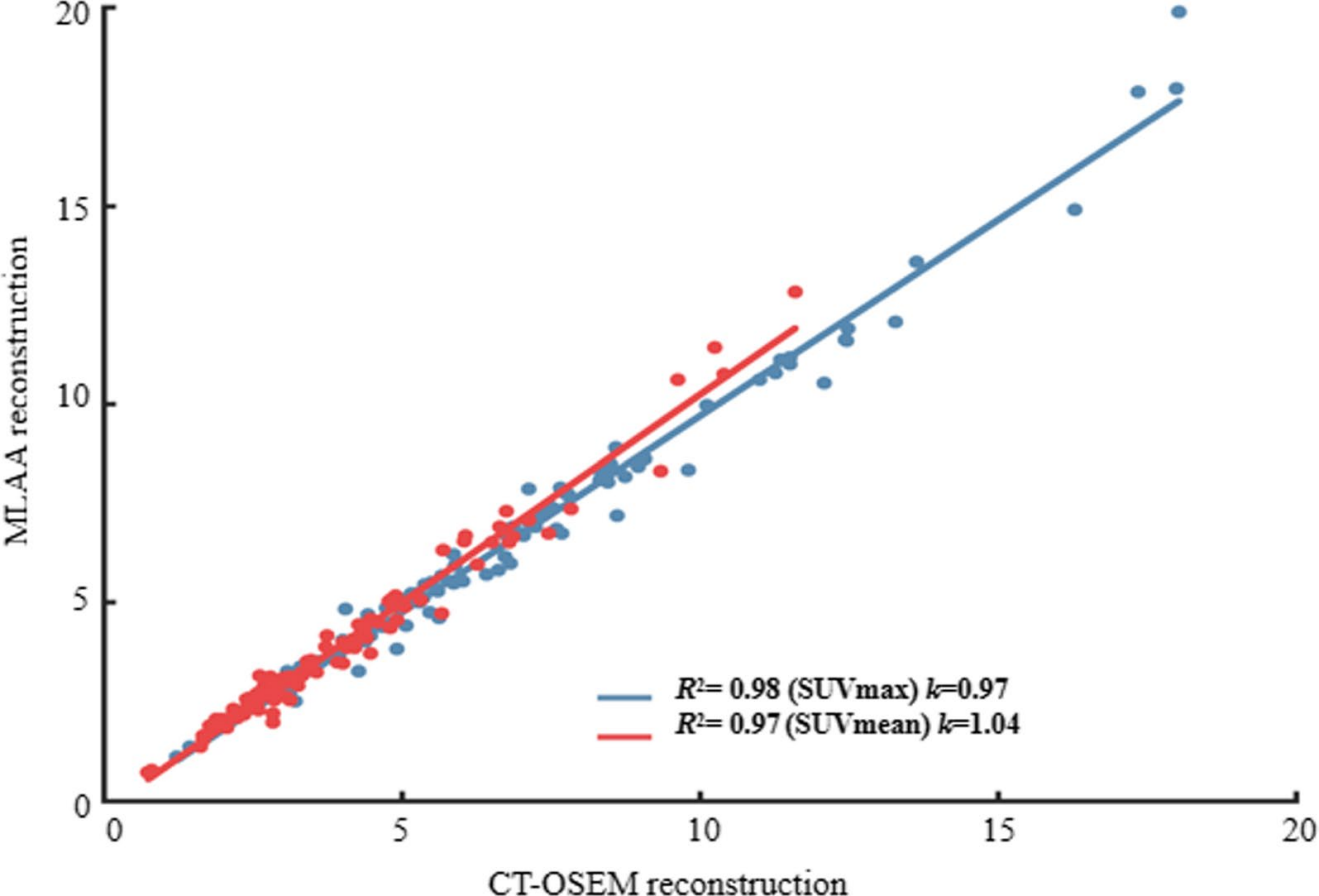
Linear regression analysis between SUVmax and SUVmean values of MLAA-based reconstruction and CT-based OSEM reconstruction in the all lesion ROIs. *R*^2^ is the correlation coefficient, *k* is the fitting slope

For the 117 delineated high uptake lesions, SUVs measured per lesion using the two reconstruction methods respectively were compared. Figure 9 shows the linear regression and correlation analysis for all the lesions. Good linear correlations between the two methods were found, with *R*^2^ = 0.98 and *k* =0.99 for SUVmax and *R*^2^ = 0.97 and *k* =1.04 for SUVmean respectively. For all the lesions, the average difference in SUVmax and SUVmean between the two methods was 7.25 ± 7.00% and 7.10 ± 7.41% respectively, showing comparable SUVs obtained using the two reconstruction methods.

For the 20 patients with identified respiration motion artifacts in the CT-OSEM images, the SUVmax and SUVmean differences between the two reconstruction methods at the two different locations in liver were reported in Table 2. At the middle part of the liver, the average SUVmax and SUVmean differences were 4.19 ± 4.23% and 4.99 ± 3.22% respectively. In comparison, the average SUVmax and SUVmean differences at the dome of the liver were 15.55 ± 15.31% and 32.79 ± 13.35% respectively.

**Table 2:** The average SUV differences between MLAA images and the reference CT-OSEM images in liver ROIs for the 20 patients with apparent respiration motion. Data represents the mean ± standard deviation

	SUVmax difference (%)	SUVmean difference (%)
Middle part	4.19 ± 4.23	4.99 ± 3.22
Dome	15.55 ± 15.31	32.79 ± 13.35

For the 10 regions with metal artifacts in the 7 patients with metal implants, the calculated average SUVmax and SUVmean differences were 52.90 ± 15.49% and 56.20 ± 13.23% respectively, suggesting significant differences in the quantification data between the two reconstruction methods when metal implants present.

## Discussion

In this study, we compared the MLAA reconstructed images to the current clinical standard OSEM reconstructed images with CT based attenuation correction. Both the phantom and patient studies showed that the MLAA reconstruction was visually and quantitatively comparable to the standard CT-OSEM reconstruction, and good correlation and consistency between the SUV measurements obtained with the two reconstruction methods were observed in areas with no evident CT and PET mismatch. For ROIs that were apparently affected by CT and PET mismatch caused by motion and metal implants, however, significant differences in image quality as well as quantification were observed between the two methods.

**Fig. 10:**
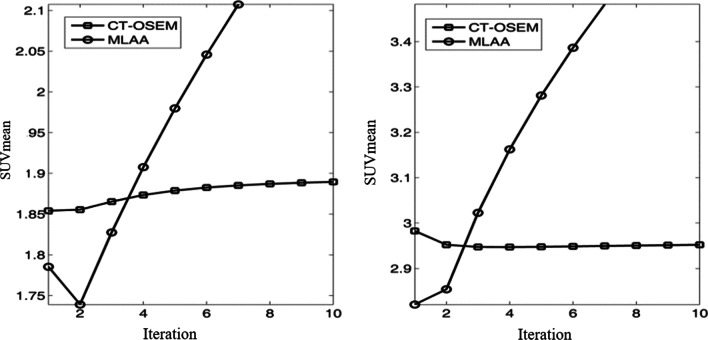
Dependency of reconstructed SUV on iteration numbers for the CT based OSEM reconstructions and MLAA based reconstructions in 2 patient studies

We have used the phantom study to determine the MLAA reconstruction parameters setting. To further verify the chosen iteration number in the reconstruction of MLAA image, we randomly selected two patients and evaluated their convergence behaviors of reconstructed SUVs against the iterations in the image reconstructions, shown in Fig. 10. A spherical volume of interest (VOI) with 3 cm diameter was placed in the liver for the two patients, and SUVmean were calculated and analyzed. Note that all the iterative reconstructions in the patient studies were performed with the subset number of 10. One can observe that the results obtained from patient study were close to the phantom studies for MLAA, shown in Fig. 1. With a known attenuation, the SUVmean in CT-OSEM image converged faster. In comparison, MLAA algorithm simultaneously estimates both the emission and attenuation image and thus is more sensitive to the noise, yielding much slower convergence. In order to reduce the potential bias in the quantitation comparison, 3 iterations were chosen for the MLAA reconstructions in the patient studies, which is in accordance with previous phantom studies. The decision optimization of the iteration number is a really crucial step before any clinical patient studies. Although a theoretical or in-depth experimental investigation of iteration number optimization is still needed for each discrete case, but it is outside the scope of this work.

In patient studies, the MLAA reconstruction provided similar results in terms of image quality as well as quantitation compared to CT-OSEM reconstruction. As shown in Table 1, for most of the organ ROIs, both the average SUVmax and SUVmean differences between the two reconstruction methods are within 10% range, suggesting comparable SUVs can be obtained using MLAA as opposed to using the conventional OSEM reconstruction with CT based attenuation correction. This is also reflected in Fig. 8 where the fitting slopes in the linear regression analysis were all close to 1. For the lung ROIs, however, the mean difference between the two methods was 10.5 ± 7.38% and 16.73 ± 8.58% for SUVmax and SUVmean respectively. This can be explained by the relatively small value of SUVs in the lung ROIs, which is in the range of 0.1–1.1. A minor absolute numerical change in SUVs could lead to a relatively large percentage difference between the two reconstruction methods. For high uptake lesions, both visual and quantitative comparisons also suggested no significant difference between the two reconstruction methods. In addition, MLAA reconstruction could also improve the PET image consistency in regions that are more affected by motion or high-density implants, whereas CT-OSEM reconstruction are affected by the mismatch between the PET and CT scans. As shown in Fig. 4, the SUV measurements were more consistent within the liver using MLAA reconstruction compared to CT-OSEM reconstruction. Also in Fig. 7, the uptake distribution exhibited consistency in the metal implant affected regions using MLAA reconstruction, whereas false positive readings were observed in the CT-OSEM reconstructed images.

As stated earlier in the last section, 20 patients were identified to have apparent respiration motion artifacts in the dome of the liver in the CT-OSEM images. Table 2 shows that, at the middle part of the liver, which is less affected by respiration, both the SUVmax and SUVmean differences between the MLAA and CT-OSEM reconstructed images were significantly smaller compared to those measured at the dome of the liver, which is more susceptible to respiration motion. To further compare the reconstructed activity distribution consistency in the liver, for both the MLAA and CT-OSEM reconstructions, we analyzed the SUV variation between the dome and the middle part of the liver per patient defined as:

2 $${\text{Liver SUV variation }}\left( {\text{\% }} \right) = \left| {\frac{{{\text{SUV }}\left( {\text{dome of liver}} \right) - {\text{SUV }}\left( {\text{middle part of liver}} \right)}}{{{\text{SUV }}\left( {\text{middle part of liver}} \right)}}} \right|{ } \times { }100{\%}$$

where the SUV at the middle part of the liver was used as the reference per patient. Table 3 shows the averages liver SUV variations for each method over the 20 patients. For the MLAA reconstruction, the average SUVmax and SUVmean variations between the dome and the middle part of the liver were 20.49 ± 9.26% and 28.00 ± 9.77% respectively. Using the CT-OSEM reconstruction, the average variations were 28.76 ± 12.90 and 42.85 ± 9.97% respectively, which were obviously larger compared to using MLAA reconstruction, further demonstrating the improved image consistency in the liver region using MLAA reconstruction.

**Table 3:** The average liver SUV variations for the 20 patients with apparent respiration motion

	SUVmax variation (%)	SUVmean variation (%)
MLAA	20.49 ± 9.26	28.00 ± 9.77
CT-OSEM	28.76 ± 12.90	42.85 ± 9.97

Data represents the mean ± standard deviation

**Fig. 11:**
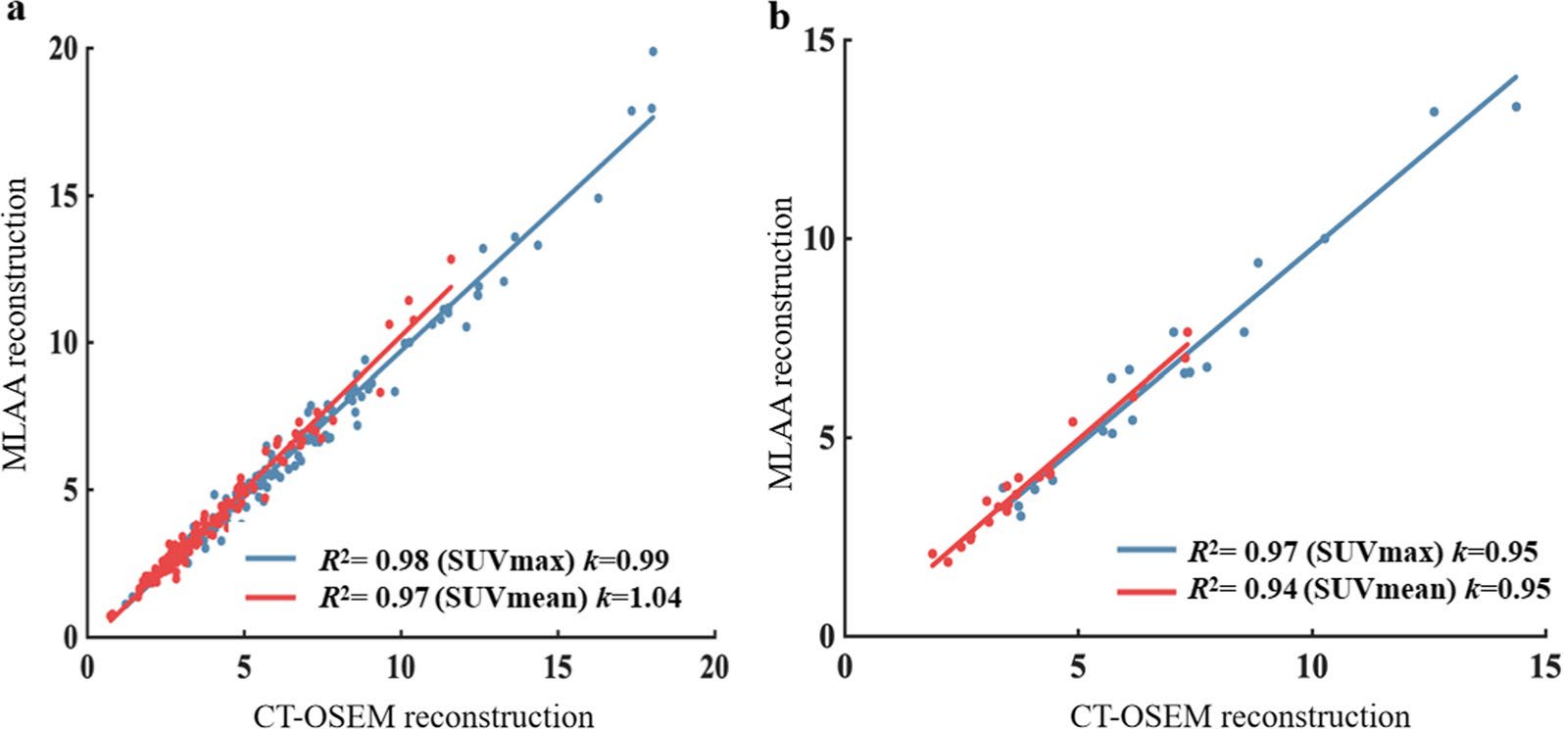
Linear regression analysis between SUVmax and SUVmean values of MLAA-based reconstruction and CT-based OSEM reconstruction in the 95 non-motion affected lesions ROIs (**a**), and 22 motion affected lesions (**b**). *R*^2^ is the correlation coefficient, *k* is the fitting slope

In this study, although 117 high uptake lesions were delineated, visual inspection found no apparent difference between the two reconstruction methods for most of the lesions. This was verified by the linear regression analysis in Fig. 9 as well as the reported average SUVmax and SUVmean differences of 7.25 ± 7.00% and 7.10 ± 7.41% respectively. To further investigate the effect of motion on the lesion quantitation, we classified the 117 lesions into motion affected group and non-motion affected group based on whether their locations could potentially be affected by respiratory motion. 22 lesions that are located within the lung base or in the liver dome were classified into the motion-affected group, while the remaining 95 lesions were grouped as non-motion affected. The SUV differences between the two reconstruction methods were calculated for these two groups of lesions and Table 4 reported the average results. For the non-motion-affected group, the average difference between the two methods was 6.23 ± 5.47% for SUVmax and 6.45 ± 5.39% for SUVmean. Linear regression analysis was also carried out and the results were plotted in Fig. 11. In the non-motion-affected group, good linear correlations between the two methods were observed, with *R*^2^ of 0.98 and 0.97, and *k* of 0.99 and 1.04 for the SUVmax and SUVmean respectively. The results suggested good consistency between the MLAA and CT-OSEM reconstructions for non-motion-affected lesions. For the motion-affected lesions, much higher average SUV differences were reported, with 11.67 ± 10.52% for SUVmax and 9.91 ± 12.77% for SUVmean respectively. A slight degradation in linear correlation were also observed in the motion-affected group, with *R*^2^ of 0.97 and 0.94, and *k* of 0.95 and 0.95 for the SUVmax and SUVmean respectively. The results suggested that for high uptake lesions affected by motion, although the two methods may not necessarily lead to visually noticeable difference in the reconstructed images, impacts on the lesion quantitation are not trivial, and using MLAA reconstruction may help to provide better quantification.

**Table 4:** The average SUV differences between MLAA images and the reference CT-OSEM images in lesions.

	SUVmax difference (%)	SUVmean difference (%)
All (117)	7.25 ± 7.00	7.10 ± 7.41
Non-motion-affected (95)	6.23 ± 5.47	6.45 ± 5.39
Motion-affected (22)	11.67 ± 10.52	9.91 ± 12.77

Data represents the mean ± standard deviation

Additionally, note that 67 patients who underwent whole-body ^18^F-FDG PET/CT scan and 117 high uptake lesions were included in this study, and we believe this data set can demonstrate the effectiveness and clinical application value of MLAA method well. Meanwhile, it is known that our clinical study on MALL algorithm with a relatively larger number of patients compared to 14 patients in ^13^N-ammonia PET/CT preclinical study [20], 12 healthy volunteers with CO_2_ stress in ^82^Rb PET/CT preclinical study [21], and 23 torso ^18^F-FDG patient clinical scans [19].

However, there are several limitations and future directions of our clinical validation studies on MLAA. First, brain applications of ^18^F-FDG PET/CT with MLAA were insufficient here, which has been discussed by Nuyts [30]. Second, datasets for patients with motion artifact in CT scan is vacant due to the fact that during the clinical acquisition process, when patients’ movement occurs in CT scan, repeated scans and terminal PET scans will be performed immediately to guarantee image quality.

## Conclusions

We evaluated and verified the performance of a MLAA-based image reconstruction method using the clinical standard OSEM reconstruction with CT-based attenuation correction as the reference. Our study demonstrated that PET images reconstructed using MLAA are clinically acceptable. In the case of no evident mismatch between CT and PET mismatch, PET images reconstructed by MLAA show comparable image qualities and the SUV values to the standard CT-based OSEM reconstruction, and it also shows good correlation and consistency between two methods. In the presence of patient motion or high-density implants in vivo, the MLAA-based reconstruction provides a more accurate tracer uptake distribution compared to the CT-based OSEM method. Our results suggest that MLAA-based image reconstruction is a useful tool in clinical practice, especially for the case when CT-based attenuation correction may cause artifacts in the reconstructed PET images.

## Data Availability

All data generated or analysed during this study are included in this published article.

## Abbreviations

PET: Positron emission tomography
CT: Computed tomography
MLAA: Maximum likelihood activity and attenuation
CT-AC: CT attenuation correction
CT-OSEM: OSEM algorithm with CT-AC
SUVmean: Mean of standardized uptake values
SUVmax: Maximum standardized uptake values
ROI: Regions of interest
TOF-PET: Time-of-flight PET
MLACF: Maximum likelihood attenuation correction factors
MLRR: Maximum likelihood reconstruction of activity and registration of attenuation
MLEM: Maximum likelihood expectation maximization
DL: Deep learning
CNN: Convolutional neural network
OSEM: Ordered subsets expectation maximization
PUMCH: Peking Union Medical College Hospital
MLTR: Maximum-likelihood for transmission
FWHM: Full width at half maximum
VOI: Volume of interest
